# Dimensioning of Biomimetic Beams under Bending for Additively Manufactured Structural Components

**DOI:** 10.3390/biomimetics9040214

**Published:** 2024-04-04

**Authors:** Tim Röver, Cedrik Fuchs, Karim Asami, Claus Emmelmann

**Affiliations:** 1 Hamburg University of Technology (TUHH), Institute of Laser and System Technologies (iLAS), Harburger Schloßstraße 28, 21079 Hamburg, Germany; karim.asami@tuhh.de (K.A.); c.emmelmann@tuhh.de (C.E.); 2 Centerline Design GmbH, Spitzenrade 3, 24107 Quarnbek, Germany; cedrik.fuchs@centerlinedesign.de

**Keywords:** beam structures, biomimetics, FEM, component design, lightweight design, parameter optimization, additive manufacturing, powder bed fusion, PBF-LB/M

## Abstract

Additively manufactured mechanical components show great lightweight characteristics and can often be enhanced by integrating biomimetic geometrical features. This study focuses on one specific subcase, namely the substitution of solid cylindrical beams that are under bending with geometrically more complex biomimetic beams. Based on the pseudo-stem of the banana plant as a role model, six geometric beam designs were derived. Given the manufacturing constraints of the PBF-LB/M process, two abstractions were selected for detailed investigation in the main part of this study. The beam lengths were set to 100 mm. Based on parametric optimization simulations, optimal design parameters were identified for the two biomimetic abstractions for 26 different bending load cases ranging from 14 to 350 Nm. Analogous parameter optimizations were performed for a solid cylindrical beam design, which was used as a reference. The results provide detailed design solutions within the investigated intervals for biomimetic beams that can be substituted into more complex mechanical component designs with ease. The analysis provides information on which structures to use for the investigated loads. With the help of the developed numerical models, designers can easily generate biomimetic beam designs for specific bending load values.

## 1. Introduction

The European Union has set the goal of developing and using sustainable mobility systems as one of the measures to achieve climate neutrality [1]. During the production of materials such as aluminum, energy is needed, and CO_2_ is emitted. One strategy to increase the sustainability of mobility systems is to reduce the amount of material used for their components through lightweight design [2]. Furthermore, lightweighting was found to be important for the reduction in fuel consumption and emissions during service, especially in applications such as aviation [3].

A study in the field of lightweight design from 2011 suggests that the utilization of biomimetic beams in structural components can improve a part’s design [4]. In their study, the authors used topology optimization to develop an initial design for a structural aircraft bracket and introduced a bamboo structure into the design in a subsequent step. The authors state that due to the material change from aluminum to titanium and the novel component design, a mass reduction of 50% compared to the previously used part could be achieved [4].

In the work by [5], a biomimetic alternative based on bamboo was developed for cylindrical shells under compression or bending that are used as mechanical components. Numerical analyses suggested that the developed biomimetic structure’s load-bearing efficiency (18.52 × 10^4^ [kN kg^−1^]) is 2.248 of that of an equal-mass, hat-stiffened cylindrical shell of the same material (8.237 × 10^4^ [kN·kg^−1^]) [5]. This example shows the great potential of the utilization of biomimetic beams in structural mechanical problems.

In [6,7], design methodologies for the integration of biomimetic beams into structural design concepts from topology optimization are presented. Mechanical topology optimization results often contain cylindrical-shaped beam structures. The works focus on the substitution of these structures by geometrically more complex biomimetic beams similar to the ones presented in [5]. As these biomimetic beams are assumed to be lighter while supporting the loads sufficiently, this approach is assumed to lead to a decrease in the mass of the overall component design [6].

Alternatively to the use of methodologies by [6,7], biomimetic beams can be used in the manual design process to enhance topology optimization results. It is common that experienced part designers and stress engineers are needed to develop component designs based on topology optimization results. Integration of biomimetic beams adds to the complexity of the design process.

The authors of this article speculate that the use of a tool for automated dimensioning of biomimetic beams can speed up the design process considerably by providing time-saving and efficient solutions for this design step. Therefore, costs may be saved while possibly achieving better technical solutions.

Due to the complex geometry of biomimetic beams such as those presented in [5], the additive manufacturing (AM) technique of powder bed fusion of metals by a laser beam (PBF-LB/M) was considered the manufacturing technique of choice in this work. This choice is in line with [4], in which the novel bracket was produced by PBF-LB/M. Furthermore, in [8] from 2020 titled “Biomimetic design and laser additive manufacturing—a perfect symbiosis?” the authors conclude that “[…] it is still obvious that both biomimetic design and LAM can benefit from each other”.

Another conclusion that is drawn in [8] is that one limitation of the combination of additive manufacturing and biomimetics is the lack of design tools for biomimetic components. This article contributes to closing this research gap by offering a design tool for biomimetic beam designs for structural components. It is noted that detailed rather than only conceptual design solutions are provided.

This article presents numerous different biomimetic beams under bending and the maximum loads that they can support. Manufacturing restrictions for PBF-LB/M were considered in the development of the designs. Therefore, the biomimetic beams presented in this article can be easily integrated into structural PBF-LB/M component designs to improve their lightweight characteristics. Furthermore, parametric optimization models can be used for the dimensioning of biomimetic beams for specific bending load values.

## 2. Methodology

In the first step, a biological blueprint was identified and abstracted to obtain four biomimetic beam designs. Considering the manufacturing constraints of PBF-LB/M, two additional biomimetic designs were developed. These were selected for a more detailed investigation in this study. For these two types of abstractions, parametric designs were developed, such that the choice of specific parameters for each parametric design was necessary to obtain the final dimensions of a biomimetic beam. Manufacturing constraints of the PBF-LB/M process were considered for the parametric designs to ensure manufacturability as well as additional design assumptions. The main focus of this part of the work was the design of the cross-sectional areas of the beams. Designs were developed so that the beam cross-sections do not change along the lengths of the beams.

The parametric biomimetic beams were then optimized using a parameter optimization with regard to bending. While doing so, optimizations were carried out for numerous specific loading conditions. The optimization goal was to minimize the cross-sectional areas of the beams (a measure that is proportional to their mass) while sufficiently supporting the loads without exceeding the yield strength of the material anywhere inside the beam. For comparison to conventional beam designs, a parametric beam design with a cross-sectional area of a circle was optimized. To ensure the validity of the simulation and optimization results, a mesh convergence analysis was carried out for one of the models. Finally, selected biomimetic beam samples were additively manufactured by PBF-LB/M in an aluminum alloy to demonstrate their manufacturability.

Therefore, the methodology of this work can be summarized as follows:

1. Abstraction of a biological blueprint and generation of four beam cross-section designs
2. Development of two abstractions and parametric models considering manufacturability by PBF-LB/M
3. Parametric optimization of two biomimetic beams under various bending loads
4. Parametric optimization of a solid cylindrical beam under various bending loads as a reference
5. Conduct of a mesh convergence study
6. Additive manufacturing of exemplary biomimetic beams

## 3. Abstraction and Parameter Optimization

In this section, parametric biological role models for beam-like structures are reviewed. Furthermore, abstractions of a biological beam structure are presented, as well as parameter optimizations and a mesh convergence analysis.

### 3.1. Biological Role Models

Apart from bamboo, numerous biological structures can be considered as role models for the design of biomimetic beams. The grass stem, porcupine quill, and hedgehog spine, which have beam characteristics, have been investigated regarding elastic buckling and showed high potential for biomimicry [9]. Grass stalks and horsetails were found to have considerable bending and distortion stiffness based on interconnected ring structures that allow for a beneficial axial second moment of area [10]. In [11], biomimetic infill structures based on a bird nest, a cocoon, trees, turtle shells, and bone were investigated. This study focused on additively manufactured samples and found great potential for the designs for the three objectives of maximal supported load in compression, resilience, and strength-to-weight ratio. Various biomimetic tubular metamaterials based on two cacti species were numerically investigated with regard to their torsional properties in [12]. In [13], biomimetic cylindrical shells were investigated numerically and experimentally with regard to their energy absorption capabilities. Designs were inspired by the biological role models of turtle, horsetail, bamboo, cattail, palm, and lemon. Lemon-inspired infill achieved the best results with regard to mean crush force and specific energy absorption [13].

Considering the study at hand focuses on the bending stiffness of biomimetic beams, grass-stalks, bamboo, and horsetail were the role models found in the literature that were of the highest relevance for this study. Figure 1 shows the cut surface of the pseudo-stem of a banana plant. The structure consists of leaves placed together, which in turn have lightweight construction characteristics: massive, heavily reinforced outer skins (epidermises), held at a distance by supports. The single layers show similarities to a sandwich structure. The overall structure shows high rigidity [14]. Due to these interesting characteristics, the authors assumed a high potential for the bending stiffness of biomimetic beams based on the banana plant pseudo-stem. Therefore, the pseudo-stem of the banana plant was analyzed in more detail in this study.

### 3.2. Abstraction of Banana Pseudo-Stem for Additive Manufacturing

The biomimetic abstractions into parametric geometrical designs are presented in the following section. In this work, the structures of the banana pseudo-stem were used as role models for the abstractions due to their high potential for mechanical applications.

Figure 2a–d show the biomimetic abstractions that were derived from the pseudo-stem. Rather than having numerous cavity rings as in the biological role model, abstractions with one cavity ring and abstractions with two cavity rings were derived. This is due to the manufacturing restrictions that were considered. Considering a minimum distance between opposing walls and minimum wall thicknesses, having more than two cavity rings would lead to beams with a rather large outer diameter that was expected to be less relevant for mechanical designs. This aspect is connected to the fact that the build envelope of a commonly used PBF-LB/M system is 500 mm × 280 mm × 365 mm [16] and comparable to other machines that are used in the AM industry.

Considering the number of cavities and the number of cavity rings, four parametric designs were derived. Three designs with one cavity ring were derived with three, four, and eight cavities, each (Figure 2a–c). For designs with one cavity ring, the one with eight cavities is the one most similar to the biological blueprint due to it having the highest number of cavities.

Furthermore, one design with two cavity rings was derived, in which the inner cavity ring consists of four cavities and the outer cavity ring consists of eight cavities (see Figure 2d).

In many AM processes, such as PBF-LB/M, support structures are necessary when parts of the design have a particular overhang angle. Support structures are a cost driver for the final part based on factors such as additional material usage, longer fabrication time, and the removal of support structures [17]. A study [18] achieved good geometrical accuracy for unsupported overhanging surfaces with an overhang angle of 45°. The samples were manufactured from AlSi10Mg [18]. Therefore, a limit overhang angle of 45° was assumed in this work. Areas that might be problematic with regard to the overhang constraint are highlighted in Figure 2a–d. Considering the main axes of the beams are parallel to the build platform (with an orientation of 0°), internal support structures would be needed for the majority of possible designs. For orientations in which the main axis is at 45–90° with respect to the build platform, no supports would be necessary within the cavities. However, it was found desirable to generate biomimetic beam designs that can be manufactured in PBF-LB/M without internal support structures, independent of their orientation in the build chamber.

Figure 2f,g show two parametric designs that were developed to generate designs that can be manufactured in any orientation by PBF-LB/M without the need for support structures within the beams (within certain intervals for each design parameter).

These structures were named “revolver drum” beams. As can be seen from Figure 2, one revolver drum beam design has one cavity ring, and the other revolver drum beam has two cavity rings. As the revolver drum structures can be manufactured more easily, they were focused on and further optimized by parameter optimization.

### 3.3. Comparison Structure

To evaluate the benefits of using biomimetic beams, a conventional structure with a cross-section of a solid cylindrical beam (SC) was used for comparison. The structure can be seen in Figure 2e.

### 3.4. Material

The aluminum alloy AlSi10Mg was chosen as the material for all parts of this work as it is widely used in lightweight applications. Materials processed by PBF-LB/M often show anisotropy. Application of a heat treatment of 2 h at 300 °C was considered for the material parameters used in this study, as a suitable heat treatment was found to reduce anisotropy [19] in AlSi10Mg. The yield strengths of horizontally and vertically manufactured AlSi10Mg samples are 141 MPa and 142 MPa, respectively. The Young’s moduli of horizontally and vertically manufactured samples are 59 GPa and 57 GPa, respectively [20]. For simplification of this study and based on the fact that the anisotropy of the material is relatively low, the material in this study was assumed to be isotopic. The material properties used for this study can be found in Table 1.

### 3.5. Parameter Optimization

The parametric biomimetic beams were optimized using a parameter optimization with regard to bending. Parametric optimizations and mesh convergence analysis were carried out in COMSOL Multiphysics 6.0 [22].

Numerous specific bending loads were considered, namely 26 different values for the bending load between 14 and 350 Nm. The optimization goal was to minimize the cross-sectional areas of the beams (as a proportional measure of their mass) while sufficiently supporting the loads without exceeding the yield strength of the material anywhere inside the beam. All optimizations were carried out considering the constant lengths of all beams of l_B_ = 100 mm.

For this work, simplifying assumptions were made. Only linear deformations and stationary loads were considered in the parametric optimizations. The more general optimization problem as implemented in COMSOL Multiphysics 6.0 can be written as

(1) $$\left.\begin{array}{c} \underset{\xi }{\min}Q\left(u\left(\xi \right),\xi \right)\\ L\left(u\left(\xi \right),\xi \right)=0\\ {lb}_{P}\leq P\left(u\left(\xi \right),\xi \right)\leq {ub}_{P}\\ {lb}_{\Psi }\leq \Psi \left(\xi \right)\leq {ub}_{\Psi }\\ {lb}_{b}\leq \xi \leq {ub}_{b} \end{array}\right\}$$

with control variables ξ, the scalar-valued objective function Q, the PDE solution u, and the discretized PDE (L(u(ξ),ξ) = 0) [23].

The constraints are divided into performance constraints on $P(u\left(\xi \right),\xi )$ that mix u and ξ, constraints on general expressions of the control variables Ψ(ξ), and constraints directly on the control variables ξ [23].

The objective functions used in this work focus on minimization of the cross-sectional areas of the beam designs and can be written as

(2) $$\left.\begin{array}{c} \underset{\xi }{\min}A\left(u\left(\xi \right),\xi \right)\\ \xi \in C \end{array}\right\}$$

with A being the cross-sectional area of the beam and C being the feasible set.

The summarized constraints for parameter optimization of the RD1 design that define the feasible set as used in this work can be written as:

(3) $$\left.\begin{array}{c} 1.5\;mm\leq r_{i,RD1}\leq 7.5\;mm\\ 1.0\;mm\leq t_{1,RD1}\leq 9.5\;mm\\ 1.0\;mm\leq t_{kmin,RD1}\leq 2\times \left(r_{i,RD1}+t_{1,RD1}\right)\\ 1.5\;mm\leq r_{k,RD1}\leq 7.5\;mm\\ 1.0\;mm\leq t_{2,RD1}\leq 9.5\;mm \end{array}\right\}$$

and

(4) $$\left.\begin{array}{c} r_{o,RD1}=r_{i,RD1}+t_{1,RD1}+t_{2,RD1}+2\times r_{k,RD1}\leq 15\;mm\\ \sigma_{max,RD1}\leq 141\;MPa \end{array}\right\}.$$

The summarized constraints for parameter optimization of the RD2 design that define the feasible set as used in this work can be written as:

(5) $$\left.\begin{array}{c} 1.5\;mm\leq r_{i,RD2}\leq 7.5\;mm\\ 1.0\;mm\leq t_{1,RD2}\leq 5.5\;mm\\ 1.0\;mm\leq t_{kmin1,RD2}\leq 2\times \left(r_{i,RD2}+t_{1,RD2}\right)\\ 1.5\;mm\leq r_{k1,RD2}\leq 7.5\;mm\\ 1.0\;mm\leq t_{2,RD2}\leq 5.5\;mm\\ 1.0\;mm\leq t_{kmin2,RD2}\leq 2\times \left(r_{i,RD2}+t_{1,RD2}+2\times r_{k1,RD2}+t_{2,RD2}\right)\\ 1.5\;mm\leq r_{k2,RD2}\leq 7.5\;mm\\ 1.0\;mm\leq t_{3,RD2}\leq 5.5\;mm \end{array}\right\}$$

and

(6) $$\left.\begin{array}{c} r_{o,RD2}=r_{i,RD2}+t_{1,RD2}+t_{2,RD2}+t_{3,RD2}+2\times r_{k1,RD2}+2\times r_{k2,RD2}\leq 15\;mm\\ \sigma_{max,RD2}\leq 141\;MPa \end{array}\right\}.$$

The summarized constraints for parameter optimization of the SC design that define the feasible set as used in this work can be written as:

(7) $$1.5\;mm\leq r_{SC}\leq 15\;mm$$

and

(8) $$\left.\begin{array}{c} r_{SC}\leq 15\;mm\\ \sigma_{max,SC}\leq 141\;MPa \end{array}\right\}$$

The constraints were developed based on the following: It was assumed that the radius of any of the cavities had to be equal to or larger than 1.5 mm to ensure powder removability in the PBF-LB process. At the same time, the radius of any cavity has to be equal to or smaller than 7.5 mm to ensure support-free manufacturability based on [24]. Furthermore, the minimum allowable wall thickness was defined as 1 mm to ensure manufacturability and additionally reduce the chances of local buckling in thin walls.

The upper limits for occurring von Mises stresses in the domains of interest are given by the yield strength of AlSi10Mg manufactured by PBF-LB, which is 141 MPa based on [20].

The constraints on the outer radii of the beams in the optimization were defined to be 15 mm. Therefore, the ratio between the length of the beams in this study (100 mm) and their maximum diameter (30 mm) would ensure a beam-like character of the design. Certain upper bounds of Equations (3) and (5) represent the maximum value for the respective parameter, considering that the remaining design parameters are set to their lower bounds while respecting a maximum diameter of the beam of 30 mm. By doing so, the solution space was further restricted to promote faster convergence of the optimization.

For parametric optimization, the popular Nelder–Mead method [25,26] as implemented in COMSOL Multiphysics 6.0 [23] was used.

The constraints according to Equations (4) and (6) were implemented using an augmented Lagrangian method based on a function implemented in COMSOL Multiphysics 6.0 based on the theory of Lagrange multipliers [23].

Parameter optimizations were conducted to find the optimal combination of design parameters for each load case, based on the information previously described. It is noted that the Nelder–Mead solver may have identified local minima of the global minimization problem. Therefore, the presented results may not be the global optimal solutions to the problems. This, however, is a common problem in non-linear optimization [27].

During each optimization simulation, multiple finite-element models were solved iteratively (based on the variation in design parameters) using automatically generated meshes. The displacement fields of all elements were chosen as quadratic serendipity to achieve results of high quality. More information regarding finite element meshes can be found in Section 3.5.

Figure 3 shows an exemplary RD1 design. The total lengths of the beam structures in the optimizations were 101 mm. However, the relevant lengths of the beams that were investigated were 100 mm. In the following, this matter is explained in more detail, and the boundary conditions and evaluation domains are also presented.

The models were divided into three domains:

- Domain 1: 0 mm ≤ z < 1 mm
- Domain 2: 1 mm ≤ z ≤ 100 mm
- Domain 3: 100 mm < z ≤ 101 mm

The beams were mechanically fixed at one end by fixing the respective circular end surface. For the application of a bending moment, the end surface of the beam on its other side was subjected to a shear force in the negative x-direction (orthogonal to the main axis of the beams).

Von Mises stresses (see Equations (4) and (6)) in the models were evaluated in domain 2. The respective volume for which von Mises stresses were evaluated is highlighted in Figure 3. Consequently, stresses were not evaluated close to the perfectly stiff fixations as well as close to the surfaces to which the force was applied. This prevents the physically meaningless evaluation of stress peaks that are due to the discretization of the mechanical problem.

At the same time, the highest stresses of interest that are expected to appear in the regions at z = 1 mm are 100 mm from the surface of the application of force. Therefore, the relevant length of the investigated beam is 100 mm. The applied forces at the free ends were varied, with values of forces ranging from 140 N to 3500 N, corresponding to 14 Nm and 350 Nm bending loads, respectively. Therefore, 26 variations for each of the parametric designs (RD1, RD2, and SC) were calculated. Based on the lever (100 mm), the maximum bending moments for each model inside the evaluated volume are given in the tables in Appendix A.

### 3.6. Discretization

To ensure low mesh-dependency of the optimization results, a mesh convergence analysis of an exemplary design was carried out. All meshes were developed using the same methodology and functions as implemented in COMSOL Multiphysics 6.0 [22]. The meshing methodology as well as the mesh convergence study are presented below. Figure 4 shows the finite-element mesh of an exemplary RD2 model. The development of the meshes for both parametric optimizations was conducted by:

1. Automatic meshing of the end surface using triangular elements (highlighted surface in Figure 4) based on five parameters for automatic meshing. The parameters were maximum element size, minimum element size, maximum element growth rate, curvature factor, and resolution of narrow regions.
2. Development of prism elements based on triangular mesh on the surface and the swept function. The thickness of the prism elements in domains 1 and 3 was chosen to be 0.25 mm (4 layers). For the rest of the beam, the thickness of the prisms was chosen to be 3 mm (33 layers).

The values of the previously mentioned meshing parameters can be found in Table 2. The values for maximum element growth rate, curvature factor, and resolution of narrow regions were defaulted in the software and were not changed. The other parameters were chosen based on a mesh convergence study, which is briefly presented below.

### 3.7. Mesh Convergence Study

The mesh convergence study was conducted with the RD2 design, as it was assumed to be the most critical one with regard to mesh dependency based on its geometric complexity. A RD2 design with the smallest possible design parameters and a downward force of 500 N at the free end was used. The parameters S_el,max_ and S_el,min_ were varied using multiplication factors of 0.5, 1, and 2. Analogously, the parameter N_PL,D1,3_ was varied using multiplication factors of 0.5, 1, and 2. Furthermore, the parameter N_PL,D2_ was varied using multiplication factors of 1/3, 1, and 3. Considering all possible combinations of these three variations, a total of 18 combinations of meshing parameters were analyzed.

The model of the mesh convergence study with the most elements (S_el,max_ = 0.5 × 2 × 10^−2^ m; S_el,min_ = 0.5 × 2 × 10^−4^ m; N_PL,D1,3_ = 2 × 4; N_PL,D2_ = 3 × 33) was considered the reference model for the other combinations, as it was assumed to be the most accurate. Its total displacement at the free end was evaluated as 4.77 × 10^−4^ m, and a maximum stress in domain 2 of 79.2 MPa was evaluated. The maximum absolute relative error of the displacement at the free end of all 17 models in this study with respect to the reference model was 0.069%. The maximum absolute relative error of the maximum stress in domain 2 of all 17 models in this study with respect to the reference model was 6.394%. The model with medium multiplication factors (S_el,max_ = 1 × 2 × 10^−2^ m; S_el,min_ = 1 × 2 × 10^−4^ m; N_PL,D1,3_ = 1 × 4; N_PL,D2_ = 1 × 33) showed relative errors for the displacement at the free end and that of the maximum stress in domain 2 of 0.006% and 2.235%, respectively.

Considering the influence of the number of elements in the finite element mesh on the computation time and the accuracy of the results of the numerical model, a balance had to be found. From the mesh convergence study, it was concluded that medium multiplication factors were a good choice for the parameter optimizations and were, therefore, used in this work. Research data from the mesh convergence study was made available in the Supplementary Materials.

## 4. Results

In this section, the numerical results of the parameter optimization and their evaluation are presented. Additionally, results regarding additive manufacturing of exemplary designs of RD1 and RD2 are presented.

### 4.1. Numerical Results

**Figure 5:**
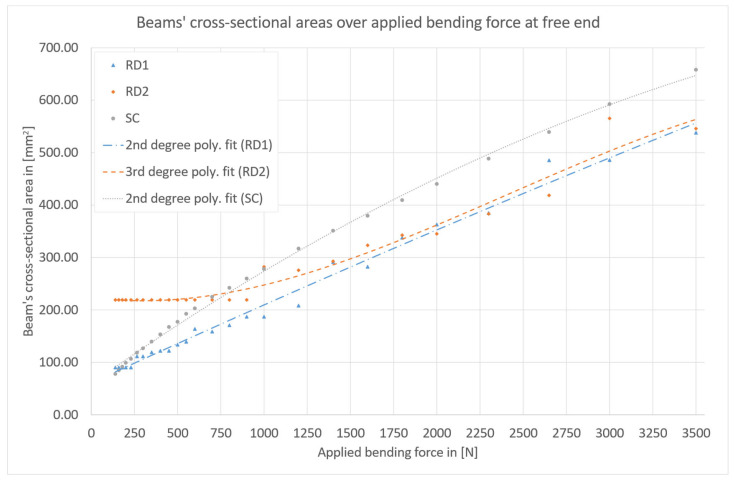
Results of the numerical parameter optimization study: Cross-sectional area over applied bending force at the free end for revolver drum beam 1 (RD1, blue), revolver drum beam 2 (RD2, orange), and solid cylindrical beam (SC, grey).

For each parametric design and investigated bending force value, one set of design parameters was identified as optimal by parametric optimization using the developed models. These optimal sets of geometric parameters for each case are listed in the tables in Appendix A. It could be confirmed that the occurring stresses did not exceed the material’s yield strength in any of the numerically investigated models. For an efficient overall component design, it has to be decided whether RD1, RD2, or SC should be used to substitute a beam. Figure 5 summarizes the results of the parameter optimization. The graph shows the cross-sectional areas of the beams over the applied bending forces. Considering lightweight engineering, a low cross-sectional area is beneficial.

All three curves have an upward trend, as expected. The curves of biomimetic beams RD1 and RD2 have a plateau for low bending force values. This is due to the fact that the minimum possible dimensions of the designs successfully support numerous of the small-valued load cases. This effect is stronger for RD2 than for RD1. It can be seen that for low bending forces up to 160 N (16 Nm), an SC beam is favorable. For bending forces between 180 N (18 Nm) and 1800 N (180 Nm), RD1 is favorable. Between 2000 N (200 Nm) and 3500 N (350 Nm), the data does not allow for a general statement on whether RD1 or RD2 is more favorable. However, the preferable design between the two can be selected for a certain load case based on Figure 5.

Therefore, using Figure 5 and the tables in Appendix A of this article, an optimized beam for component design can be realized with little effort for the investigated load cases.

Polynomial fits in Figure 5 support the previous statements that for low force values, SC beams are favorable, for medium force values, RD1 beams are beneficial, and for high force values, optimized RD1 and RD2 beams should be compared to identify the lighter beam.

TIn the following, characteristics of designs RD1, RD2, and SC are discussed: In general, regarding bending loads, the distance of material from the neutral axis in the cross-section of a beam is important. The relevant measure is the axial second moment of area [28]. Therefore, the solid cylinder is a rather disadvantageous design for bending loads since most of the material is located close to the neutral axis. Comparing designs RD1 and RD2 to the SC design, it is beneficial that RD1 and RD2 have cavities at their centers as well as additional cavity rings such that considerable amounts of mass are moved away from the neutral axis, thereby increasing the axial second moment of area.

Based on the geometrical constraints that were defined, the SC design allows for the smallest diameter of the three designs as well as the smallest cross-sectional area and smallest axial second moment of area. At the same time, it allows for the largest cross-sectional area and the largest axial second moment of area at the maximum diameter defined for the designs. However, the lightweight design character of the design with respect to bending loads is rather low. Designs RD1 and RD2 are more strongly constrained. The intervals for minimum and maximum cross-sectional area and minimum and maximum axial second moment of area, indirectly given by the geometrical constraints, are shorter than those of the SC parametric design. At the same time, it was expected that RD1 and RD2 would give better results under bending than SC beams. The results (see Figure 5) confirm that for most load values, RD1 and RD2 designs indeed provide solutions with better lightweight character than the SC designs.

**Figure 6:**
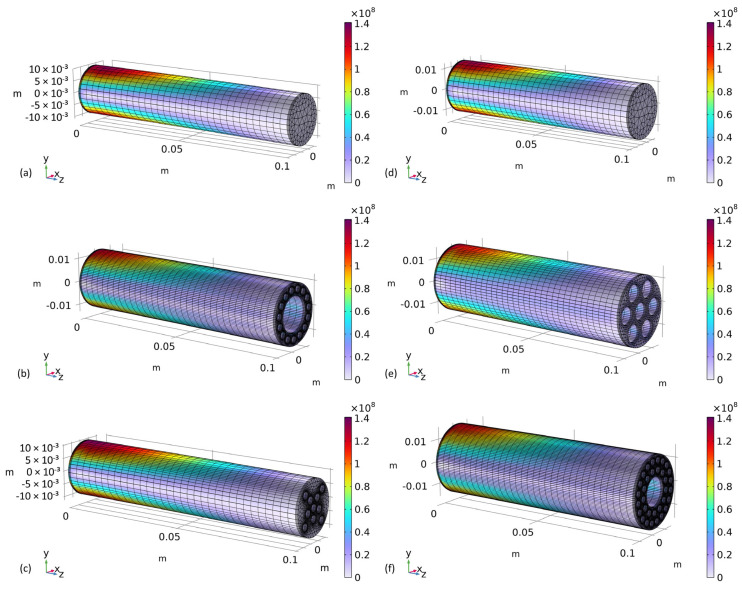
Von Mises stresses in exemplary optimization results. (**a**) SC optimized for 1200 N; (**b**) RD1 optimized for 1200 N; (**c**) RD2 optimized for 1200 N; (**d**) SC optimized for 2300 N; (**e**) RD1 optimized for 2300 N; (**f**) RD2 optimized for 2300 N.

Figure 6 shows the von Mises stresses in six exemplary optimization results. The relevant design parameters were taken from the optimization results, as stated in the tables in Appendix A. It can be seen that for all models, high stresses occur close to the supported ends, as was expected based on beam theory. Furthermore, it can be seen that all exemplary models show stresses close to the yield strength of the material. This is beneficial with regard to lightweight engineering and the optimization objective. If the maximum stress within a model were considerably below the yield strength of the material, this would suggest a rather low lightweight character of the beam.

Both SC designs (see Figure 6a,d) are well in line with expectations. The highest stresses occur near the supported ends of the beams (at a large distance from the downward-facing forces) and at the largest distances from the neutral axes of the beams. Both RD1 designs (see Figure 6b,e) give good results regarding their cross-sectional area compared to SC and RD2 designs (see Figure 5), while it is striking that the ratio between parameters r_i,RD1_ and r_k,RD1_ is considerable for the two results. Based on these unintuitive designs and parameters as optimization results, the authors conclude that parametric optimization can help product designers generate efficient and novel beam designs for unique load cases. With regard to the exemplary RD2 designs shown in Figure 6c,f, it can be seen that they also exhibit strong differences. The large inner cavity of RD2-2300 N in Figure 6f helps to move material away from the neutral axis and achieve a great axial second moment of area. For RD2-1200 N in Figure 6c, it can be seen that the value for t_kmin2,RD2_ results in a considerable distance between the cavities of cavity ring 2 of the design. This results in bulk material without cavities at the outer regions of the cross-section and a beneficial axial second moment of area. Also, in this case, the qualitatively different results show that parameter optimization can provide unintuitive but efficient design solutions.

Further qualitative analysis of the optimization results revealed that the majority of optimized beams for RD1 and RD2 had relatively small radii for the cavities of the cavity rings and relatively low distances between the cavities of the cavity rings. Furthermore, the majority of beams had a relatively large radius for the center cavity. At the same time, numerous optimized beams differed qualitatively from this trend, as indicated by the results shown in Figure 6. For RD1, a trend could be identified of increasing radii of the cavities of the cavity ring with increasing bending loads (1400–3500 N). No qualitative design trends could be identified for the results of RD2.

Optimization simulation files of each investigated type of beam, covering each investigated type of loading, were made available in the Supplementary Materials of this article. Using these files, unique values for the bending load can be given as an input to the parameter optimizations. Furthermore, the lengths of the beams can be adapted to finally obtain customized, optimized parameters for one of the biomimetic beams. However, when the length is adapted, the number of layers of prism elements in domain 2 should be changed proportionally. The authors see great potential for the use of the provided models for systematic and reproducible biomimetic design of mechanical components in research and industry.

### 4.2. Additive Manufacturing

**Figure 7:**
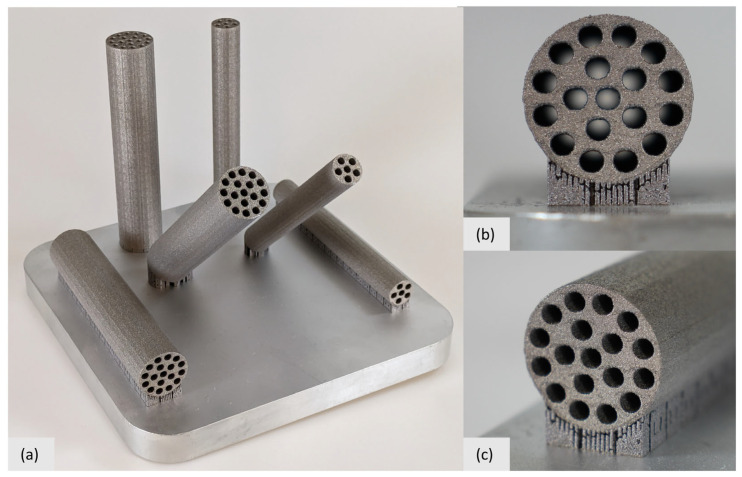
Exemplary revolver drum beams (RD1 and RD2) in three different orientations (0°, 45°, and 90°) manufactured by PBF LB/M in AlSi10Mg. The build platform has a base area of 152 mm × 152 mm. (**a**) Photograph of the entire build job; (**b**) Close-up of the RD2 design manufactured at 0°, showing that cavities are free of support structures; (**c**) Additional close-up of the RD2 design manufactured at 0°, showing no visible defects or geometric irregularities.

Figure 7 shows additively manufactured revolver drum beams. Exemplary versions of parametric designs RD1 and RD2 were produced in orientations of 0°, 45°, and 90°. Data preparation for additive manufacturing was performed with Autodesk Netfabb Ultimate slicer software (version 2023.1) [29]. Production in aluminum alloy AlSi10Mg was carried out using the PBF-LB/M system MPrint from One Click Metal [30]. The fabrication was performed with a laser power of 170 W, a scan speed of 1200 mm/s, a hatch distance of 0.1 mm, a layer thickness of 20 µm, and a focus diameter of 70 µm. It could be shown that the exemplary beam designs could be manufactured successfully in three different orientations (0°, 45°, and 90°) without the use of support structures within the cylindrical cavities of the beams. In Figure 7b, it can be seen that the internal channels of the 0°-RD2 beam could be manufactured without internal supports in the cavities. It could be confirmed that all other manufactured beams of the build job could also be manufactured without internal supports. Visual inspection revealed no defects or geometric irregularities (see Figure 7a–c). Therefore, it can be expected that designs RD1 and RD2 are well-suited for substitution in mechanical components in terms of manufacturing constraints in the PBF-LB/M process when using AlSi10Mg as a material. It is noted that powder removability has to be ensured for the powder within the cavities of the beams. With regard to the mechanical properties of the manufactured beams by PBF-LB/M, the influence of different orientations in the build volume has to be briefly considered. In that respect, it can be assumed that their orientation only has a minor influence, as relevant material parameters only show low levels of anisotropy, as previously stated.

## 5. Conclusions and Future Prospects

Additively manufactured mechanical components show great lightweight characteristics. Such component designs can often be improved by the integration of biomimetic geometric features. This work focuses on one specific subcase, namely the substitution of solid cylindrical beams that are under bending by geometrically more complex biomimetic beams.

Based on the pseudo-stem of the banana plant as a role model, six geometric beam designs were derived. Considering the manufacturing constraints of the PBF-LB/M process, two abstractions were chosen for detailed investigation in the main part of this study. Beam lengths were chosen to be 100 mm.

Based on parametric optimization simulations, optimal design parameters were identified for the two biomimetic abstractions for 26 different bending load cases with values between 14 and 350 Nm. Furthermore, analogous parameter optimizations were performed for a solid cylindrical beam design that was used as a reference.

The results give detailed design solutions within the investigated intervals for biomimetic beams that can be substituted into more complex mechanical component designs with ease.

The analysis shows that SC beams are beneficial for small bending loads, RD1 beams are beneficial for medium values, and for higher bending loads, both RD1 and RD2 designs should be considered to choose the most beneficial design. Furthermore, the results show that, in some cases, rather unintuitive values for the design parameters were obtained by the parametric optimizations. This suggests that the provided methods and optimization models have a high potential for the design of biomimetic mechanical components.

The authors made the optimization models available in the Supplementary Materials. With the help of these models, designers can easily generate optimized beam designs for SC, RD1, and RD2 for specific bending load values.

Exemplary beam designs were produced in aluminum alloy AlSi10Mg by the additive manufacturing process PBF-LB/M. It could be shown, that the designs could be manufactured successfully without support structures inside the cavities. Therefore, the designs are well-suited for use in lightweight components manufactured by additive manufacturing.

The authors plan to extend the optimization simulations so that combined load cases considering bending moments, normal forces, shear forces, and torsion forces can be considered within one model. It is noted that the way the models were set up with one fixed end and one end on which the bending force acts is beneficial for the advancement that is planned for the optimization models. Furthermore, parameter optimizations should be developed for the remaining four abstractions presented in this study.

**Figure 8:**
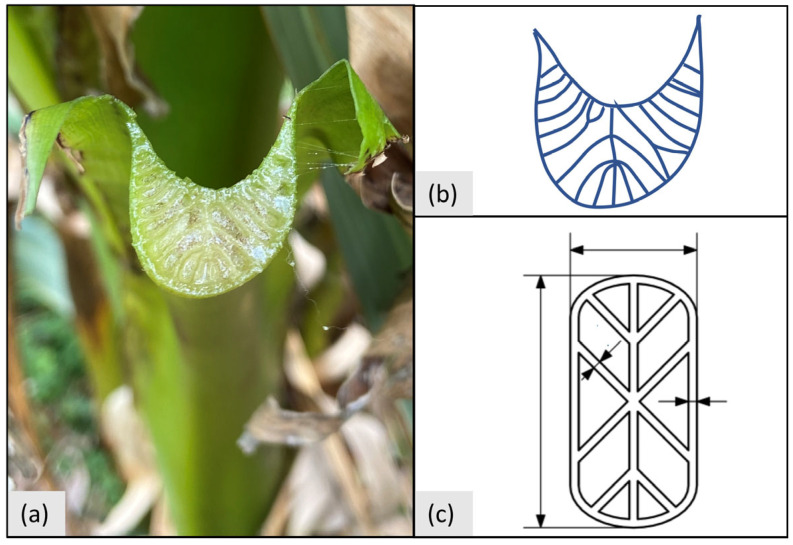
Exemplary abstraction of banana plant petiole cut surface: (**a**): Photograph; (**b**): Contour line; (**c**): Abstraction.

Furthermore, additional parametric beam designs should be developed analogously to allow for a greater solution space. Common beam designs such as I-beams and circular tubes should be investigated in future works, as well as the development of parametric beam designs based on 3D topology optimization results. Additionally, other biological blueprints, such as grass stalks, bamboo, horsetail, and the banana plant petiole, could be used for the development of parametric beam designs. The banana plant petiole could be of special interest because of the inner structures that are at approximately 45° and, therefore, well in line with overhang manufacturing constraints in the PBF-LB/M process (see Figure 8).

## Figures and Tables

**Figure 1:**
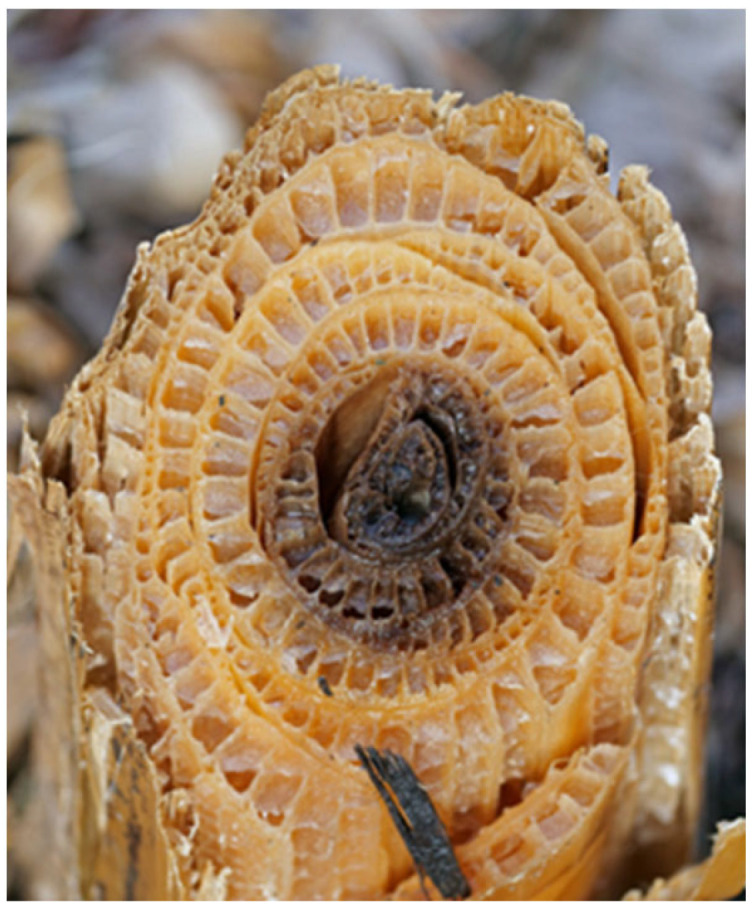
Photograph of the cut surface of the pseudo-stem of a banana plant. Reprinted from [15].

**Figure 2:**
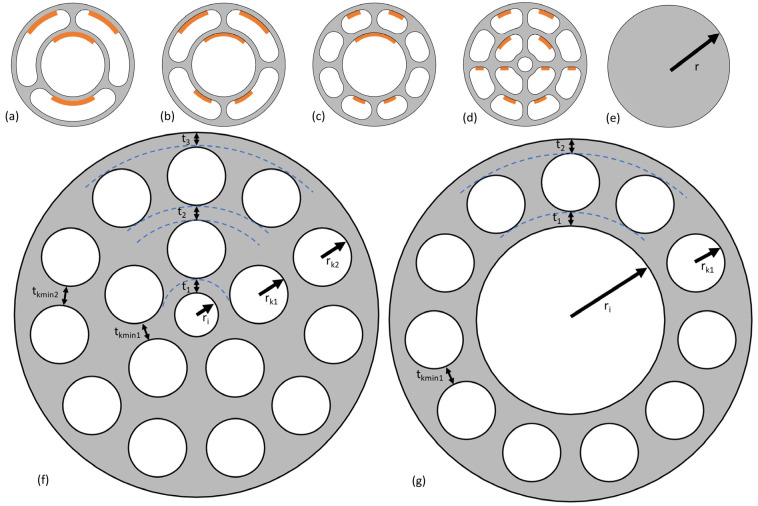
(**a**–**d**) Intermediate biomimetic abstractions of the banana plant pseudo-stem in the form of parametric cross-sectional designs of beams. Problematic areas considering overhang manufacturing constraints by PBF-LB/M are highlighted in orange; (**e**) Solid cylindrical beam; (**f**,**g**) Biomimetic abstractions of banana plan pseudo-stem adapted for manufacturing by PBF-LB/M, were named “revolver drum” beams.

**Figure 3:**
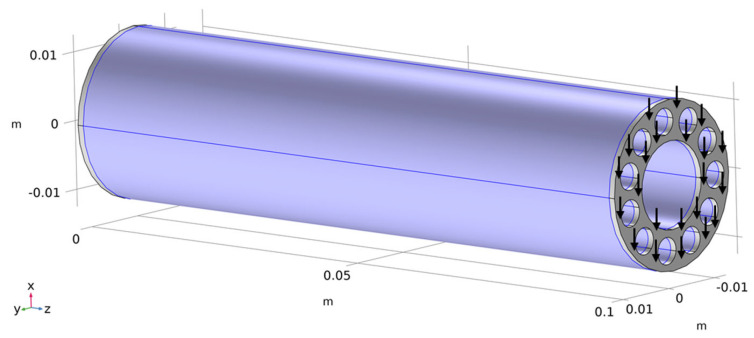
Exemplary optimization model with volume considered for stress evaluation highlighted. The model was mechanically fixed at z = 0 m, and a boundary force was applied to the free end.

**Figure 4:**
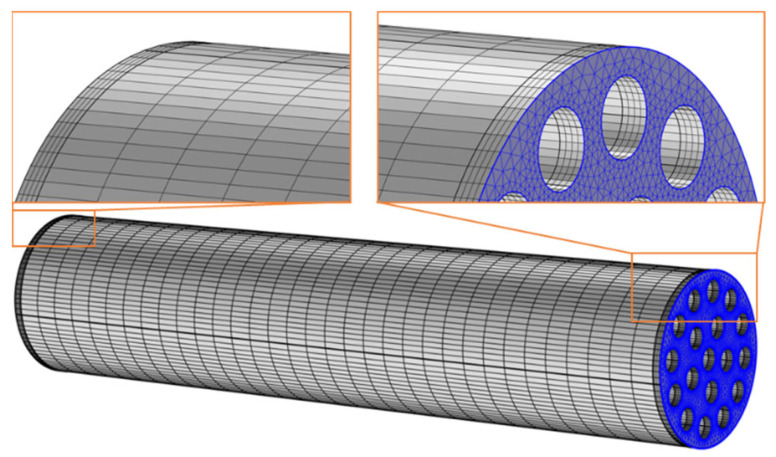
Finite-Element mesh of an exemplary RD2 model.

**Table 1:** Material properties of heat-treated AlSi10Mg manufactured by PBF-LB.

Material Property	Value
Yield strength	141 MPa [20]
Young’s modulus	57 GPa [20]
Poisson’s ratio	0.34 [21]
Density	2.67 g/cm^3^ [20]

**Table 2:** Meshing parameters.

Meshing Parameter	Value
Maximum element size (S_el,max_)	2 × 10^−2^ m
Minimum element size (S_el,min_)	2 × 10^−4^ m
Maximum element growth rate	1.3
Curvature factor	0.2
Resolution of narrow regions	1.0
Number of prism element layers in domains 1 and 3 (N_PL,D1,3_)	4
Number of prism element layers in domain 2 (N_PL,D2_)	33

## Data Availability

Data are contained within the article and the Supplementary Materials of this article.

## Supplementary Materials

The following supporting information [31] can be downloaded at: https://doi.org/10.15480/882.9113, Portable Document Format file S0: Readme of dataset (file name: 0_Readme.pdf); COMSOL Multiphysics 6.0 file S1: Parameter optimization of RD1 under bending by 140–700 N (file name: 1_RD1_bending_140–700.mph); COMSOL Multiphysics 6.0 file S2: Parameter optimization of RD1 under bending by 800–3500 N (file name: 2_RD1_bending_800–3500.mph); COMSOL Multiphysics 6.0 file S3: Parameter optimization of RD2 under bending by 140–700 N (file name: 3_RD2_bending_140–700.mph); COMSOL Multiphysics 6.0 file S4: Parameter optimization of RD2 under bending by 800–3500 N (file name: 4_RD2_bending_800–3500.mph); COMSOL Multiphysics 6.0 file S5: Parameter optimization of SC under bending by 140–3500 N (file name: 5_SC_bending_140–3500.mph); COMSOL Multiphysics 6.0 file S6: Mesh Convergence Study (file name: 6_RD2_mesh_con_parametric_sweep.mph); Portable Document Format file S7: Mesh convergence evaluation overview (file name: 7_mesh_con_evaluation_overview.pdf).

## Nomenclature

A	Cross-sectional area of beam
C	Feasible set
L(u(ξ),ξ) = 0	System of discretized partial differential equations
l_B_	Length of beams (constant), 100 mm
lb_b_	Lower bound of control variables
lb_P_	Lower bound of performance constraint
lb_Ψ_	Lower bound of general expressions of the control variables
N_PL,D1,3_	Number of prism element layers in domains 1 and 3 (meshing)
N_PL,D2_	Number of prism element layers in domain 2 (meshing)
P(u(ξ),ξ)	Performance constraints
*Q*	Scalar-valued objective function
r	Radius
r_i_	Inner radius
r_k1_	Radius of cavities in first cavity ring
r_k2_	Radius of cavities in second cavity ring
r_o_	Outer radius
S_el,max_	Maximum element size (meshing)
S_el,min_	Minimum element size (meshing)
t_kmin1_	Minimum distance between cavities in first cavity ring
t_kmin2_	Minimum distance between cavities in second cavity ring
t_1_	Wall thickness of first ring
t_2_	Wall thickness of second ring
t_3_	Wall thickness of third ring
*u*	PDE solution
ub_b_	Lower bound of control variables
ub_P_	Upper bound of performance constraint
ub_Ψ_	Upper bound of general expressions of the control variables
x_P1_	Arbitrary design parameter P_1_
x_P2_	Arbitrary design parameter P_2_
x_Pn_	Arbitrary design parameter P_N_
ξ	Control variables
*σ_max_*	Maximum allowable von Mises stress
Ψ(ξ)	General expressions of the control variables
…_RD1_	Subscript indicating reference to revolver drum beam 1
…_RD2_	Subscript indicating reference to revolver drum beam 2
…_SC_	Subscript indicating reference to solid cylindrical beam

## Appendix A

**Table A1:** Parameter optimization results for revolver drum 1 under bending loads.

Revolver drum 1 (RD1)				Model parameters									
				Young’s modulus				Poisson’s ratio			Density		Length
				57 [GPa]				0.34			2.67 [g/cm^3^]		100 [mm]
				Design variables									
				Parameter			Variable			Minimum		Maximum	
				Inner radius			r_i_			1.5 [mm]		7.5 [mm]	
				Wall thickness first ring			t_1_			1 [mm]		9.5 [mm]	
				Wall thickness second ring			t_2_			1 [mm]		9.5 [mm]	
				Radius of cavities in cavity ring			r_k1_			1.5 [mm]		7.5 [mm]	
				Minimum distance between cavities in cavity ring			t_kmin1_			1.0 [mm]		2 × (r_i_ + t_1_)	
				Constraints									
				Physical constraint			Numerical constraint			Minimum		Maximum	
				Yield strength of AlSi10Mg			Maximum von Mises stress in domain 2			-		141 [MPa]	
				Maximum diameter of 30 mm of beam			r_i_ + t_1_ + t_2_ + 2 × r_k1_			-		15 [mm]	
Bending													
Force [N]	Max. Bending Moment [Nm]	r_i_ [mm]	t_1_ [mm]		t_2_ [mm]	r_k1_ [mm]			t_kmin1_ [mm]			Area [mm^2^]	
140	14	1.5000	1.0000		1.0000	1.5000			1.0000			90.32	
160	16	1.5000	1.0000		1.0000	1.5000			1.0000			90.32	
180	18	1.5000	1.0000		1.0000	1.5000			1.0000			90.32	
200	20	1.5000	1.0000		1.0000	1.5000			1.0000			90.32	
230	23	1.5000	1.0000		1.0000	1.5000			1.0000			90.32	
265	26.5	1.5000	1.0000		1.0000	1.5000			5.0000			111.53	
300	30	1.5000	1.0000		1.0000	1.5000			5.0000			111.53	
350	35	3.0936	1.0000		1.0000	1.5000			1.0000			119.18	
400	40	3.1825	1.0000		1.0000	1.5000			1.0000			121.97	
450	45	3.1825	1.0000		1.0000	1.5000			1.0000			121.97	
500	50	3.7825	1.0000		1.0000	1.5000			1.0000			133.75	
550	55	4.1825	1.0000		1.0000	1.5000			1.0000			139.25	
600	60	5.1825	1.0000		1.0000	1.5000			1.0000			163.60	
700	70	5.0255	1.0000		1.0000	1.5000			1.0000			158.66	
800	80	5.6387	1.0000		1.0000	1.5000			1.0000			170.86	
900	90	6.3787	1.0000		1.0000	1.5000			1.0000			187.04	
1000	100	6.3784	1.0000		1.0000	1.5000			1.0000			187.03	
1200	120	7.5000	1.0000		1.0000	1.5000			1.0000			208.13	
1400	140	1.8643	3.4934		1.2866	2.9185			1.2589			291.18	
1600	160	6.4023	1.2577		1.2781	1.9665			2.1404			282.34	
1800	180	4.2807	2.0517		2.0384	2.2948			1.3703			337.77	
2000	200	4.0351	2.1814		2.1598	2.7252			1.1168			362.80	
2300	230	3.7896	1.9188		1.5913	3.9043			1.7545			384.65	
2650	265	2.8336	2.9109		2.2365	3.0961			4.4002			485.41	
3000	300	1.7899	2.1242		3.5810	3.8958			1.0000			485.67	
3500	350	1.5000	1.0000		1.0000	7.5000			1.0000			537.99	

**Table A2:** Parameter optimization results for revolver drum 2 under bending loads.

Revolver drum 2 (RD2)						Model parameters								
						Young’s modulus			Poisson’s ratio		Density		Length	
						57 [GPa]			0.34		2.67 [g/cm^3^]		100 [mm]	
****						Design variables								
						Parameter			Variable		Minimum		Maximum	
						Inner radius			r_i_		1.5 [mm]		7.5 [mm]	
						Wall thickness first ring			t_1_		1 [mm]		5.5 [mm]	
						Wall thickness second ring			t_2_		1 [mm]		5.5 [mm]	
						Wall thickness third ring			t_3_		1 [mm]		5.5 [mm]	
						Radius of cavities in first cavity ring			r_k1_		1.5 [mm]		7.5 [mm]	
						Radius of cavities in second cavity ring			r_k2_		1.5 [mm]		7.5 [mm]	
						Minimum distance between cavities in first cavity ring			t_kmin1_		1 [mm]		2 × (r_i_ + t_1_)	
						Minimum distance between cavities in second cavity ring			t_kmin2_		1 [mm]		2 × (r_i_ + t_1_ + 2 × r_k1_ + t_2_)	
						Constraints								
						Physical constraint			Numerical constraint		Minimum		Maximum	
						Yield strength of AlSi10Mg			Maximum von Mises stress in domain 2		-		141 [MPa]	
						Maximum diameter of 30 mm of beam			r_i_ + t_1_ + t_2_ + t_3_ + 2 × r_k1_ + 2 × r_k2_		-		15 [mm]	
	Bending													
Force [N]	Max. Bending Moment [Nm]	r_i_ [mm]	t_1_ [mm]	t_2_ [mm]	t_3_ [mm]		r_k1_ [mm]	r_k2_ [mm]		t_kmin1_ [mm]		t_kmin2_ [mm]		Area [mm^2^]
140	14	1.5000	1.0000	1.0000	1.0000		1.5000	1.5000		1.0000		1.0000		219.13
160	16	1.5000	1.0000	1.0000	1.0000		1.5000	1.5000		1.0000		1.0000		219.13
180	18	1.5000	1.0000	1.0000	1.0000		1.5000	1.5000		1.0000		1.0000		219.13
200	20	1.5000	1.0000	1.0000	1.0000		1.5000	1.5000		1.0000		1.0000		219.13
230	23	1.5000	1.0000	1.0000	1.0000		1.5000	1.5000		1.0000		1.0000		219.13
265	26.5	1.5000	1.0000	1.0000	1.0000		1.5000	1.5000		1.0000		1.0000		219.13
300	30	1.5000	1.0000	1.0000	1.0000		1.5000	1.5000		1.0000		1.0000		219.13
350	35	1.5000	1.0000	1.0000	1.0000		1.5000	1.5000		1.0000		1.0000		219.13
400	40	1.5000	1.0000	1.0000	1.0000		1.5000	1.5000		1.0000		1.0000		219.13
450	45	1.5000	1.0000	1.0000	1.0000		1.5000	1.5000		1.0000		1.0000		219.13
500	50	1.5000	1.0000	1.0000	1.0000		1.5000	1.5000		1.0000		1.0000		219.13
550	55	1.5000	1.0000	1.0000	1.0000		1.5000	1.5000		1.0000		1.0000		219.13
600	60	1.5000	1.0000	1.0000	1.0000		1.5000	1.5000		1.0000		1.0000		219.13
700	70	1.5000	1.0000	1.0000	1.0000		1.5000	1.5000		1.0000		1.0000		219.13
800	80	1.5000	1.0000	1.0000	1.0000		1.5000	1.5000		1.0000		1.0000		219.13
900	90	1.5000	1.0000	1.0000	1.0000		1.5000	1.5000		1.0000		1.0000		219.13
1000	100	1.6138	1.0000	1.0000	1.0000		1.5000	1.5000		1.0000		10.7700		282.11
1200	120	1.5000	1.0000	1.0000	1.0000		1.5000	1.5000		1.1546		7.0727		275.67
1400	140	1.8967	1.0353	1.0000	1.4824		1.6326	1.5815		1.0182		1.5059		292.63
1600	160	3.3163	1.1083	1.0000	1.0000		1.5000	1.5000		1.3440		2.9104		323.18
1800	180	5.1825	1.0000	1.0000	1.0000		1.5000	1.5000		1.0000		1.0000		342.54
2000	200	5.3522	1.0000	1.0000	1.0000		1.5000	1.5000		1.0000		1.0000		345.07
2300	230	6.4000	1.0000	1.0000	1.0000		1.5000	1.5000		1.0000		1.0000		383.12
2650	265	7.4000	1.0000	1.0000	1.0000		1.5000	1.5000		1.0000		1.0000		418.46
3000	300	1.5250	1.0000	1.0000	5.4000		1.5000	1.5000		1.0083		1.0083		565.26
3500	350	5.3117	1.6190	1.0019	1.3417		1.8887	1.9413		1.7177		2.0912		545.67

**Table A3:** Parameter optimization results for the solid cylindrical beam under bending loads.

Solid cylindrical beam (SC)			Model parameters							
			Young’s modulus	Poisson’s ratio			Density			Length
			57 [GPa]	0.34			2.67 [g/cm^3^]			100 [mm]
			Design variables							
			Parameter		Variable			Minimum	Maximum	
			Inner radius		r			1.5 [mm]	15 [mm]	
			Constraints							
			Physical constraint		Numerical constraint			Minimum	Maximum	
			Yield strength of AlSi10Mg		Maximum von Mises stress in domain 2			-	141 [MPa]	
			Maximum diameter of 30 mm of beam		r			-	15 [mm]	
Bending										
Force [N]	Max. Bending Moment [Nm]	r [mm]				Area [mm^2^]				
140	14	4.9805				77.93				
160	16	5.1914				84.67				
180	18	5.4023				91.69				
200	20	5.6133				98.99				
230	23	5.8242				106.57				
265	26.5	6.1406				118.46				
300	30	6.3516				126.74				
350	35	6.6680				139.68				
400	40	6.9844				153.25				
450	45	7.3008				167.45				
500	50	7.5117				177.27				
550	55	7.8281				192.51				
600	60	8.0391				203.03				
700	70	8.4609				224.90				
800	80	8.7773				242.03				
900	90	9.0938				259.80				
1000	100	9.4102				278.19				
1200	120	10.0430				316.86				
1400	140	10.5700				351.01				
1600	160	10.9920				379.59				
1800	180	11.4140				409.29				
2000	200	11.8360				440.10				
2300	230	12.4690				488.42				
2650	265	13.1020				539.25				
3000	300	13.7340				592.60				
3500	350	14.4730				658.03				
